# 5-Fluorouracil Treatment of CT26 Colon Cancer Is Compromised by Combined Therapy with IMMODIN

**DOI:** 10.3390/ijms23126374

**Published:** 2022-06-07

**Authors:** Vlasta Demeckova, Dagmar Mudronova, Sona Gancarcikova, Peter Kubatka, Karol Kajo, Monika Kassayova, Bianka Bojkova, Marian Adamkov, Peter Solár

**Affiliations:** 1 Department of Animal Physiology, Institute of Biology and Ecology, Faculty of Science, P. J. Šafárik University in Košice, 040 01 Košice, Slovakia; vlasta.demeckova@upjs.sk (V.D.); monika.kassayova@upjs.sk (M.K.); bianka.bojkova@upjs.sk (B.B.); 2 Department of Microbiology and Immunology, University of Veterinary Medicine and Pharmacy, 041 81 Košice, Slovakia; dagmar.mudronova@uvlf.sk (D.M.); sona.gancarcikova@uvlf.sk (S.G.); 3 Department of Medical Biology, Jessenius Faculty of Medicine, Comenius University in Bratislava, 036 01 Martin, Slovakia; kubatkap@gmail.com; 4 Department of Pathology, St. Elisabeth Cancer Institute, 812 50 Bratislava, Slovakia; karol.kajo@ousa.sk; 5 Department of Histology and Embryology, Jessenius Faculty of Medicine, Comenius University in Bratislava, 036 01 Martin, Slovakia; marian.adamkov@uniba.sk; 6 Department of Cell Biology, Institute of Biology and Ecology, Faculty of Science, P. J. Šafárik University in Košice, 040 01 Košice, Slovakia; 7 Department of Medical Biology, Faculty of Medicine, P. J. Šafárik University, SNP1, 040 11 Košice, Slovakia

**Keywords:** Immodin, 5-FU, colon cancer, CT26-cancer model

## Abstract

Due to the physiological complexity of the tumour, a single drug therapeutic strategy may not be sufficient for effective treatment. Emerging evidence suggests that combination strategies may be important to achieve more efficient tumour responses. Different immunomodulators are frequently tested to reverse the situation for the purpose of improving immune response and minimizing chemotherapy side effects. Immodin (IM) represents an attractive alternative to complement chemotherapy, which can be used to enhance the immune system after disturbances resulting from the side effects of chemotherapy. In the presented study, a model of CT26 tumor-bearing mice was used to investigate the effect of single IM or its combination with 5-fluorouracil (5-FU) on colon cancer cells. Our results highlight that the beneficial role of IM claimed in previous studies cannot be generalised to all chemotherapeutic drugs, as 5-FU toxicity was not increased. On the contrary, the chemotherapeutic anti-cancer efficacy of 5-FU was greatly compromised when combined with IM. Indeed, the combined treatment was significantly less effective regarding the tumour growth and animal survival, most probably due to the increased number of tumour-associated macrophages, and increased 5-FU cytotoxic effect related to kidneys and the liver.

## 1. Introduction

Despite a dramatic increase in our understanding of the epidemiology, molecular biology, and clinical aspects of colorectal cancer (CRC) over the past several decades, it is still the third most diagnosed malignancy, with 1.8 million new cases confirmed annually worldwide [1]. The 2020 estimates reveal that over 341,000 people in the EU-27 are diagnosed with CRC (12.7% of all cancer diagnoses), and it represents the second most common cause of death (12.4% of all cancer deaths) [2]. Although multidisciplinary care is used to treat localized colon cancer, systemic chemotherapy is the mainstay of CRC therapeutic strategies. The fluoropyrimidines, especially 5-fluorouracil (5-FU), have been widely used for over 60 years in the treatment of a range of cancers; however, it is in CRC that 5-FU has had the greatest impact [3]. It is an antimetabolite drug that prevents DNA and RNA synthesis [4] and is routinely combined with other chemotherapies to improve patients’ survival [5]. An increased understanding of the mechanism of the action of 5-FU has led to the development of strategies that increase its anti-cancer activity to 40–50%. However, despite advances, drug resistance remains a significant limitation to the clinical use of 5-FU [3]. Moreover, the other main side effects of 5-FU are neuro/hematological toxicity and hemorrhagic enteritis [6]. Different chemotherapeutic agents, including 5-FU [7], also activate a diverse range of pro-inflammatory pathways. Thus a strong tumour-associated immune response is often initiated by cancer therapy resulting in the development/worsening of distinct histopathological changes in intestinal mucosa [8]. Although the inflammatory reactions can also exert beneficial effects in enhancing anti-tumour immunity, modulating the level of therapy-induced inflammation may improve cancer outcome and treatment. IMMODIN^®^ (IM; lyophilized leukocyte-derived dialysate from healthy human donors) is a heterogeneous mixture of biologically active immunomodulating agents (also known as Transfer Factor-TF or Dialyzable Leukocyte Extract-DLE), which has no reported side effects or toxicity [9,10,11]. Therefore, IM can represent a promising alternative to complement chemotherapy and can be used to modulate the IS after disturbances resulting from the side effects of chemotherapy drugs, including 5-FU. As there is no report about the effect of IM on colon cancer in vivo, our study was undertaken to explore the effects of both single IM and its combination with 5-fluorouracil on the tumour growth, hematological parameters, and survival of CT26 tumour-bearing mice. CT26 cells share molecular features with aggressive, undifferentiated, refractory human colorectal carcinoma cells; therefore, CT26 is one of the most extensively used syngeneic mouse tumour models in drug development for human colorectal cancer. Combination therapies using 5-FU with appropriate immunomodulators may enhance the clinical benefit of 5-FU and improve its outcomes in colorectal cancer treatment.

## 2. Results

### 2.1. Tumour Weight

**Figure 1:**
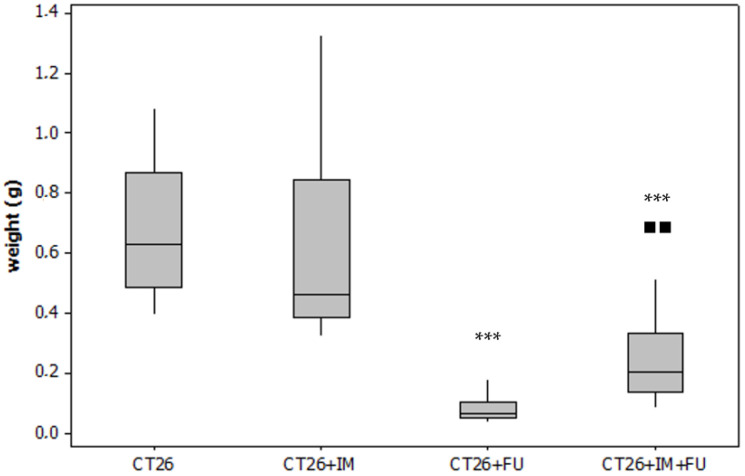
The effect of treatments on tumour weight. CT26-tumour control (*n* = 10); CT26 + IM-CT26 treated with IM (*n* = 10); CT26 + 5-FU-CT26 treated with 5-FU (*n* = 10); CT26 + IM + 5-FU-CT26 treated with IM and 5-FU (*n* = 10). *** *p* < 0.001 vs. CT26 group, ■■ *p* < 0.01 vs. CT26 + 5-FU.

Whereas single IM treatment had no effect on tumour growth, 5-FU treatment resulted in a significant decrease in tumour weight (*p* < 0.001) (Figure 1) compared to the untreated CT26 group. The combination treatment (5-FU + IM) also inhibited tumour growth (*p* < 0.001) compared to CT26 control; however, the efficacy of the chemotherapeutic drug was significantly (*p* < 0.01) compromised (Figure 1).

### 2.2. Animal Survival

**Figure 2:**
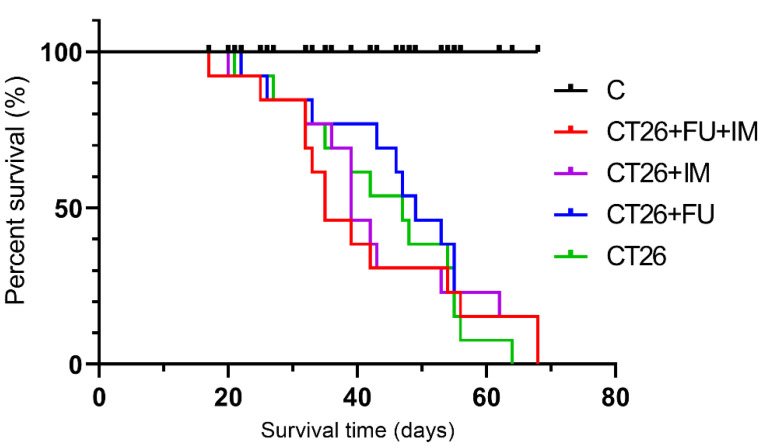
Effect of the treatments on animal survival. Kaplan-Meier survival curves (*n* = 15) were compared using the log-rank test (Mantel-Cox); No significant difference in lifespan was observed between treated CT26 mice *p* = 0.0312 (CT26 vs. CT26 + IM), *p* = 0.0019 (CT26 vs. CT26 + 5-FU), *p* < 0.0007 (CT26 vs. CT26 + IM + 5-FU); C, untreated control; IM, IM treatment; CT26, tumour control; CT26+ IM, CT26 treated with IM; CT26 + 5-FU, CT26 treated with 5-FU; CT26 + IM + 5-FU, CT26 treated with IM and 5-FU.

Each experimental group of tumour-bearing mice had a decreased survival compared to the control group (*p* < 0.001). No significant differences in lifespan were observed between treated experimental groups compared to the untreated CT26 group, but the fact that the efficacy of a chemotherapeutic drug was compromised by IM was also confirmed by a trend of survival curves (Figure 2). By day 40, there were less than 40% of animals left in the group treated with combination therapy (CT26 + 5-FU + IM), while more than 75% survival was observed in the CT26 + 5-FU group and about 60% in the untreated cancer control group (CT26). The median survival time for animals that did not undergo any treatment (CT26) was 47 days, compared to 49 days (HR = 0.72; 95% CI, 0.3–1.5) and 39 days (HR = 0.89; 95% CI, 0.4–1.9) for the CT26 + 5-FU and CT26 + IM groups, respectively. The combined treatment has led to the shortest median survival time of 35 days (HR = 0.97; 95% CI, 0.4–2.1) (Figure 2).

### 2.3. Analysis of Hematological Parameters

Tumour growth was associated with marked leukopenia and in the blood of tumour bearing animals (CT26) compared to untreated controls (C) (*p* < 0.01) (Table 1). Induction of cancer resulted in significantly lower numbers of lymphocytes (*p* < 0.001), but a higher percentage of the granulocyte count (*p* < 0.001) compared to a healthy control group (C). Therapy with 5-FU had the greatest impact on the normalization of leukocyte numbers, but its effect was weakened in combined treatment with IM. The neutrophil-lymphocyte ratio (NLR) calculated from the white cell differential count revealed a significant increase (*p* < 0.001) in the CT26 group compared to the control (Table 1). 5-FU treatment normalized both NLR values to the level of healthy animals (Table 1), but its effect was slightly compromised by IM. Thrombocytopenia was also observed in all tumour bearing animals. However, IM alone accelerated the platelet reduction, which was reflected in significantly lower thrombocyte numbers (*p* < 0.05) compared to the CT26 group. Similar results were observed in red blood cell parameters, where 5-FU treatment alone normalized erythrocyte numbers, hematocrit, or hemoglobin concentration without any additional/compromising effect of IM (Table 1).

### 2.4. Serum Biochemical Parameters

To point out the cytotoxicity of 5-FU and IM therapy in vivo, we monitored selected serum biochemical parameters (Table 2).

CT26 tumour induction caused alteration of liver enzymes. None of the treatments affected decreased serum ALP concentrations, but in terms of AST/ALT parameters, 5-FU was the most efficient treatment to normalize their concentrations (Table 2). Similar to hematological analyses, the application of IM in combination with 5-FU affected the normalising efficacy of a chemotherapeutic drug. No significant differences were observed in the concentration of total proteins and serum albumin. However, elevated creatine levels normalized by 5-FU treatment were demonstrated. Moreover, there was an increased urea concentration in all tumour groups (*p* < 0.01), with the highest level recorded in the case of combination treatment (*p* < 0.001 vs. C; *p* < 0.01 vs. CT 26). The concentrations of total cholesterol were not affected except for combined treatment, where significantly higher concentration (*p* < 0.05) compared to healthy controls (C) and the CT26 group (*p* < 0.01) was shown (Table 2). Although tumour induction increased both HDL and LDL cholesterols (*p* < 0.001), all treatments showed a normalizing effect only on LDL cholesterol levels compared to the untreated CT26 group. Tumour induction also increased triglycerides (TG), although the values were still at the physiological level (Table 2).

### 2.5. Flow Cytometric Analysis of a Single-Cell Suspension from Tumour Tissue

**Figure 3:**
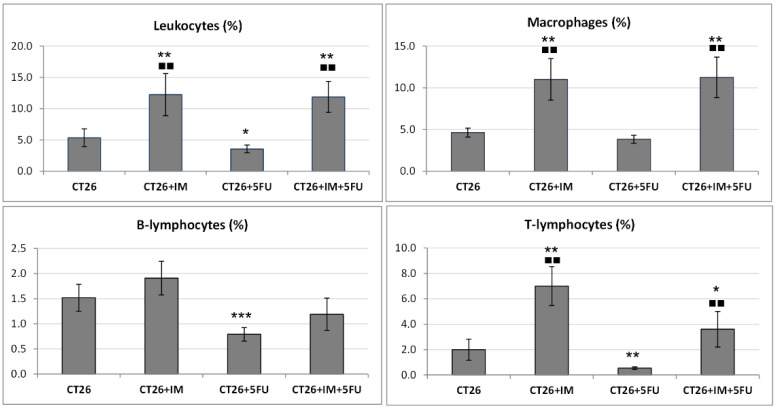
Immune cell proportions (%) in the tumour microenvironment. Data are expressed as mean percentage ± SD; * *p* < 0.05, ** *p* < 0.01, *** *p* < 0.001 vs. CT26; ^■■ ^*p* < 0.01 vs. CT26 + 5-FU; CT26- tumour control (*n* = 10); CT26 + IM-CT26 treated with IM (*n* = 10); CT26 + 5-FU-CT26 treated with 5-FU (*n* = 10); CT26 + IM + 5-FU-CT26 treated with IM and 5-FU (*n* = 10).

The results of the percentage of immune cells in the tumour microenvironment are shown in Figure 3. The percentage of total tissue leukocytes, macrophages, and T-lymphocytes increased significantly in the groups CT26 + IM and in combination treatment (CT26 + IM + 5-FU) compared to the CT26 group. On the other hand, 5-FU chemotherapy treatment significantly reduced total leukocytes (*p* < 0.05), T (*p* < 0.01), and B lymphocytes (*p* < 0.001) compared with a cancer control group (Figure 3).

### 2.6. Immunohistochemical and Histopathological Analyses of Primary Tumour Tissue

**Figure 4:**
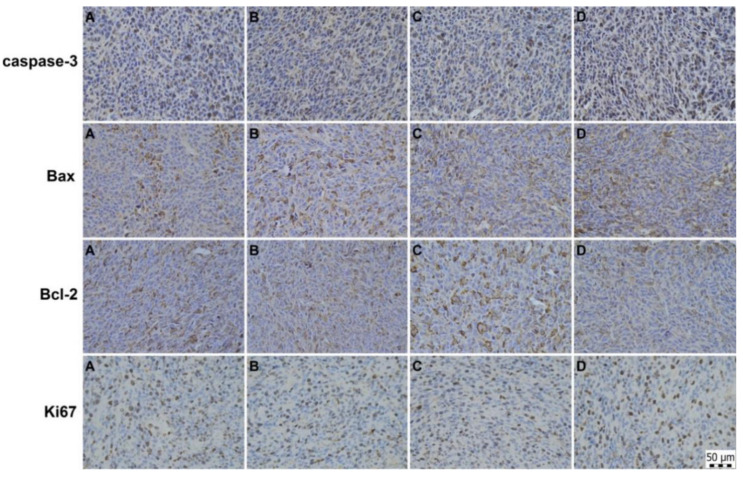
Representative images of caspase-3, Bax, Bcl-2, and Ki67 protein expressions in CT26 mouse colon carcinoma cells in vivo after the treatment with IM, 5-FU, and combination IM + 5-FU. (**A**) CT26, (**B**) CT26 + IM, (**C**) CT26 + 5-FU, (**D**) CT26 + IM + 5-FU. Polyclonal caspase-3 antibody (Bioss, Woburn, MA, USA), polyclonal Bax and Bcl-2 antibodies (Santa Cruz Biotechnology, Paso Robles, CA, USA), and monoclonal Ki67 antibody (Dako, Glostrup, Denmark) were used. Final magnifications in all photos: ×400.

An immunohistochemical analysis of cancer cells in vivo showed significant proapoptotic effects of single IM and 5-FU as well as the combination of IM + 5-FU (Figure 4, Table 3). Compared to controls, the expression of caspase-3 was increased by 7% (*p* > 0.05) in IM group, by 11.5% (*p* < 0.05) in the 5-FU group, and by 10% (*p* < 0.05) in IM + 5-FU treatment. Evaluating Bax/Bcl-2 ratio, additive proapoptotic effects of combined treatment compared to alone treatments were observed. In this regard, IM increased the Bax/Bcl-2 ratio by 29% (*p* < 0.05), 5-FU by 62% (*p* < 0.001), and combined treatment by 104.5% (*p* < 0.001) when compared to control group. Ki67, a prominent proliferation marker, was not changed in treated groups vs. controls (Figure 4; Table 3).

Within the histopathological analysis, we have evaluated a total of 14 tumours/allografts (control group, *n* = 4; IM, *n* = 4; 5-FU, *n* = 2; IM + 5-FU, *n* = 4). The assessing of necrosis/all tumour area ratio (N/A) showed following data: CT26-20.6%, CT26 + IM-31.1%, CT26 + 5-FU-0.0%, CT26 + IM + 5-FU-12.2%. Histopathological evaluation of CT26 tumours did not reveal any changes in MAI between control and treated groups (Table 3); this result is in accordance with the data gained from the immunohistochemical analysis (Ki67 marker).

## 3. Discussion

Due to the physiological complexity of the tumour, a single drug therapeutic strategy may not be sufficient for effective treatment. Moreover, a major concern for anti-cancer drugs is their potential toxicity and immunosuppression. Therefore, different immunomodulators are frequently tested to reverse the situation to improve immune response and minimize chemotherapy side effects. However, antagonistic activities and conflicting pharmacokinetics of co-administered agents can potentially reduce therapeutic activity. Since IM is the product of T-lymphocytes, the main mechanism of action is at the level of cell-mediated immunity [12]. T-lymphocytes are fundamental for immune reaction, particularly concerning solid tumours. The gastrointestinal tract contains several phenotypically and functionally distinct populations of T cells, which may play an important role in anti-tumour immunity [13,14]. As cancer has been associated with a T-cell dysfunction [15], the clinical use of IM could represent a promising adjuvant treatment in cancer therapy. IM includes both inducer fractions and regulator fractions. Inducer fractions transport a mature immune response from donor to recipient. Regulator fractions help control overreactions and stimulate the IL-10 formation and inhibitory cytokines by Th2 cells [16]. Recent proteomic characterization of IM identified 48 unique proteins associated with blood cells or plasma [17]. The highest number of proteins was related to innate immunity and to inflammatory response, including those having the potential to inhibit cytokines and treat the cytokine storm. IM also contains proteins that can speed up recovery by regulating cell growth and repair. IM as a treatment has been shown to improve cellular immunity in patients with immune deficits [18] and even increase the quality of cancer patients’ lives during chemotherapeutic treatments [19]. The results obtained in our previous study highlighted a potentially beneficial role for IM in alleviating paclitaxel-induced toxicity during breast cancer therapy [20]. However, the present study emphasizes the important fact that the beneficial role of IM cannot be generalized to all chemotherapeutic drugs, as the anti-cancer efficacy of the fluoropyrimidine 5-FU was greatly compromised when combined with IM. Indeed, the combined treatment of IM and 5-FU was significantly less effective in slowing down CT26 tumour growth and animal survival compared with animals treated with a single 5-FU. 5-FU’s multi-factorial mechanisms affecting the immune system were recently reviewed by Gmeiner et al. [21]. In this regard, 5-FU modulates the host anti-tumour response by affecting multiple cell types [22]. For example, 5-FU may cause some tumour cells to be more visible to the adaptive immune system [23], or it may be cytotoxic to immunosuppressive immune cells [24]. However, 5-FU also activates processes that are disruptive to potential anti-tumour immune response, e.g., it causes gut inflammation [25] or alters the composition of the gut microbiome [26]. A repeated cycle of 5-FU therapy tends to repress the anti-tumour immune functions as well. The drug initially promotes proliferation and cytotoxicity of tumour-infiltrating cytotoxic T cells after one cycle of treatment, but after repeated cycles, the anti-tumour immune functions get impaired, with the release of immune-suppressive factors such as TGF-ß and IL-10 [27]. Moreover, the tumour microenvironment and immune reaction, including different cytokines, have an important role in the regulation and modulation of bodily response to 5-FU therapy [28,29]. A protumourigenic microenvironment is characterized by an increased infiltration of tumour-associated macrophages (TAM), where their presence is strongly associated with tumour progression, therapy resistance, and poor survival rates [30]. TAMs directly stimulate cancer cell proliferation through the secretion of epidermal growth factor (EGF) [31], promote tumour angiogenesis by vascular EGF (VEGF) secretion [32], and remodel the ECM by secreting metalloproteinases (MMPs) [33]. The sensitivity of cancer patients to 5-FU therapy was regulated by the TAMs due to secretion of CCL22 and decreased apoptosis induced by 5-FU [29]. Based on the significantly higher numbers of macrophages observed in the tumour tissue of animals after combined treatment, we can assume that this could be one reason for the decreased effectiveness of 5-FU in our experimental model. Since IM in our study could increase the levels of peripheral monocytes, these results suggest that the combination of chemotherapeutic and immunomodulatory compounds must be chosen carefully to avoid reduced therapeutic activity. It is clear that tissue-resident-macrophage expansion and new monocyte recruitment are critical for developing multiple solid cancers [34]. This suggests that the different monocyte recruiting pathways enable tumour survival by supplying the tumour microenvironment with pro-tumourigenic macrophages. Thus, suppression of monocyte/macrophage recruitment is a potential therapeutic strategy to eliminate or reduce their involvement in tumourigenesis.

However, co-administration of IM in our study resulted in the opposite action. Although combined treatment increased the percentage of infiltrating T-cells, it could be the consequence of increased TAMs, which can recruit regulatory T cells (Tregs) by secreting the chemokine CCL2 resulting in enhancing the immunosuppressive CRC microenvironment [35]. Moreover, T cells isolated from tumours often show signs of exhaustion and have distinct metabolic signatures [36]. As tumours progress, the immune infiltrate changes dramatically in abundance and composition. Indeed, while the elimination and equilibrium phases are generally dominated by CD8 + T cells, type 1 Th lymphocytes and NK cells, escape is accompanied by a massive decrease of immune infiltrate or by the accumulation of cells that inhibit tumour-targeting immune responses, including regulatory T cells and immunosuppressive myeloid cells (MDSC) [37].

While some studies reported that 5-FU decreased immunosuppressive myeloid and Treg cell populations consistent with enhancing anti-tumour immunity, 5-FU also was shown to activate the inflammasome in dying myeloid cells leading to IL-1β secretion that increased angiogenesis and stimulated tumour growth. Using depletion experiments and knock-out mice, it was shown that the MDSC-derived IL1-β triggers IL17 production by CD4+ T cells, limiting the anti-cancer efficacy of 5-FU [38]. In our study, using the CT26/Balb-c model, multiple cycles of 5-FU decreased the proliferation of cytotoxic T-cells specific to CT26, indicating the potential for 5-FU to attenuate anti-tumour immunity by inhibiting the proliferation of tumour-specific T-cell populations [27]. Therefore, higher infiltration of T cells in the tumour tissue of animals treated by combined treatment (5-FU + IM) could represent immunosuppressive T cells which further compromised 5-FU efficacy.

It would be interesting to perform a follow-up study with a more robust immunophenotyping analysis of tumor tissue by including markers for different T-lymphocyte subtypes. Initial myeloablative effects of 5-FU stimulate a rebound response that tends to restore normal levels of immune cells [39]. While this rebound effect of a single high dose 5-FU treatment was observed in mice, the effects of multiple treatments simulating clinical regimens are more complex. Studies using the CT26/Balb-c model demonstrated that multiple cycles of 5-FU treatment improved tumour growth inhibition better than a single cycle but did not improve survival [27]. This is also in agreement with our study where multiple doses of 5-FU didn’t improve animal survival compared to the untreated CT26 group, and its co-administration with IM caused an even further reduction of survival time. A study with the same CT26/Balb-c model [27] reported that immune cell sub-populations of blood did not differ between treated and control groups except for a significant reduction in B-cells with multiple treatments. We obtained a similar observation, where, despite significant leukopenia induced by tumour induction, no difference between the levels of leukocytes in animals treated with 5-FU compared to the healthy control was observed. Although we obtained significantly lower levels of peripheral lymphocytes compared to healthy animals, their numbers were still the highest of all treated groups. The significant decrease of peripheral lymphocytes was associated with the rise of granulocytes in all tumour-bearing groups, indicating an inflammatory response involving systemic alterations. While lymphocyte count reflects cell-mediated immunity, systemic inflammation can be reflected by neutrophilia. It has been reported that high NLR together represent not only an easily measurable and inexpensive marker of systemic inflammation [40] but in CRC patients, it has been implicated with adverse oncological outcomes and associated with worse overall survival [41,42]. In our experiment, 5-FU significantly lowered the NLR ratio, which may contribute to anti-tumour response. The slight decrease in the lymphocyte count after 5-FU, also confirmed by our flow cytometric analysis of spleen tissue, could result from B-cell reduction [27]. The administration of cytotoxic agents has always been complicated with many adverse effects, such as liver injury [43], which eventually increases serum concentrations of aminotransferases. 5-FU is associated with a low rate of transient serum aminotransferase elevations during therapy and has been implicated in rare cases of clinically apparent acute liver injury [44]. In our study, the liver injury was induced by CT26 tumour growth, possibly due to metastatic processes, and was reduced by 5-FU treatment.

We did not observe any additional adverse effects of 5-FU on liver tissue. However, its co-administration with IM significantly increased the cytotoxic effect of 5-FU on liver tissue. The liver injury is believed to be associated with hepatocyte apoptosis [45] which is modulated by mitogen-activated protein kinases (MAPKs) that transduce extracellular signals to various subcellular compartments regulating a variety of biologic processes, e.g., cell survival and apoptosis, inflammation, and necrosis [46,47]. Bcl-2 family members, including proapoptotic protein Bax and anti-apoptotic protein Bcl-2, have also been extensively studied as regulators of apoptosis [48]. The histopathological results of the present study showed that IM in combination with 5-FU increased the expression of the proapoptotic protein Bax and decreased levels of the anti-apoptotic protein Bcl-2, thus possibly also elevating apoptosis of the hepatic cells. Bax and Bcl-2 are the major members of the Bcl-2 family whose potential roles in tumour progression and prognosis of different malignancies have been of interest in various studies during the last decade. Whereas Bax promotes cell death through permeabilization of the mitochondrial outer membrane, Bcl-2 prevents apoptosis by inhibiting the activity of Bax [49]. Conflicting results have been reported in the case of introducing Bax expression as an independent prognostic and predictive marker of colorectal cancer. A significant correlation between longer survival and increased Bax expression in tumour cells has been observed in previous studies [50,51]. However, in other studies, down-regulation of Bax and up-regulation of Bcl2 expressions were associated with better survival, respectively [51,52]. These results became more confusing when the therapeutic status of patients was considered [53]. It has been shown that in patients after surgery, high Bax expression is associated with improved survival. However, patients with a lack or low Bax expression in their tumour cells, but not those with high expression, benefit from 5-FU-based adjuvant therapies. Although many studies have been done on the prognostic significance of counteracting Bcl-2 and Bax proteins, most of them failed to find a significant relationship between Bcl-2 expression levels and clinicopathological parameters of colorectal cancer. A possible explanation for these results can be the presence of other members of the Bcl-2 family, which can act independently from Bcl-2 [54].

Abnormalities in cellular metabolism have been reported in many types of cancers. Metabolic reprogramming contributes to tumour progression and metastasis and is considered an important hallmark of cancer [55]. Lipids are essential nutrients for cells, acting as the structural components of cell membranes, signaling molecules and energy suppliers. Abnormal lipid metabolism is an important metabolic phenotype in CRC cells, involved in a wide range of colorectal carcinogenesis, progressions, and metastases [56]. Upregulated lipogenic enzymes are frequently found in patients with aggressive metastatic CRC [57], implying that lipid metabolism-based therapy by targeting these enzymes might be a novel therapeutic option for CRC. Increased lipid availability allows cancer cells to overcome growth inhibition checkpoints and apoptotic signaling, leading to enhanced survival, proliferation, and morphological changes required for invasion and metastasis [58]. In our study, IM + 5-FU co-treatment significantly elevated total cholesterol, which could also contribute to the worst overall performance of animals on combined treatment as higher circulating cholesterol may feed CRC progression.

Moreover, activation of toll-like receptors (TLR) by high cholesterol levels can promote an inflammatory state that may contribute to tumour progression [59]. Knowing that cholesterol is essential for malignant cell survival, HDL-mediated cholesterol trafficking could play a significant role in tumour development [60]. Changes in HDL levels, HDL particle distribution, and HDL receptors and modulators have been consistently found in CRC patients [61]. HDL is, so far, the most complex lipoprotein particle, and it is now clear that its role both in health and in disease also includes an antioxidant, anti-inflammatory, immunomodulatory, anti-apoptotic, and anti-thrombotic functions [62]. Several studies have shown that HDL particles can stimulate the growth of breast cancer cells in vitro, as well as increase the aggressiveness of malignant tumours in mice [63,64]. In terms of immunomodulatory function, HDL prevents the conversion of macrophages to the pro-inflammatory M1 phenotype [65], thus decreasing the pro-inflammatory environment, which is critical for anti-tumour immune responses. HDL cholesterol in our study was significantly elevated in all cancer groups, and none of the treatments could induce its decrease back to the normal values.

Further studies are needed to establish whether the association of HDL with CRC development is causal or merely a reflection of disturbed metabolic balance in this disease. The anti-cancer drug 5-FU has a narrow therapeutic range, and its effects are dose- and concentration-dependent [66]. 5-FU undergoes extensive metabolism with intracellular activation to cytotoxic fluoronucleotides, mainly in tumour tissue, followed by an efficient multistep catabolism in the liver so that only a small amount of 5-FU (approximately 10% of the dose) is eliminated unchanged by the kidneys [67]. The catabolites of 5-FU are excreted predominantly by the kidneys and are expected to accumulate in renal failure. In our study, however, we did not observe the 5-FU toxicity on renal function in the case of a single treatment, but its cytotoxic effect was significantly increased during co-treatment with IM. Over the last four decades, 5-FU therapeutic drug monitoring has made significant progress. There is now a validated algorithm of 5-FU dose adjustment based on plasma 5-FU levels to reduce toxicity and improve the efficacy of 5-FU [66]. However, our biochemical results suggest that the combination of chemotherapeutic and immunomodulatory compounds must be chosen carefully to ensure that our effort to develop a more effective cancer-killing treatment is not associated with the promotion of a protumourigenic environment in cancer tissue.

## 4. Materials and Methods

The State Veterinary and Food Administration of the Slovak Republic approved the experimental protocol number 4296/12-221e, and the animals were handled and sacrificed humanely in accordance with the guidelines established by the relevant commission and the Directive 2010/63/EU of the European Parliament and the Council on the protection of animals used for scientific purposes.

### 4.1. Reagents

5-Fluorouracil (5-FU, Sigma-Aldrich, St. Louis, MO, USA) was administered intraperitoneally at a concentration of 0.4 mg/mouse. IMMODIN^®^ (IM) was purchased from Imuna Pharm a.s. (Šarišské Michaľany, Slovakia). Drugs were freshly prepared on the day of use. IM was prepared by dissolving the lyophilized dialysate of 200 × 10^6^ leukocytes in 4 mL water for injection according to the manufacturer’s instruction.

### 4.2. Cell Line

CT26, a colon carcinoma cell line generated from BABL/C mice, was purchased from the American Type Tissue Culture Collection (ATCC, Manassas, VA, USA) and was cultured in RPMI-1640 medium supplemented with 5 mM glutamate and 10% inactivated fetal bovine serum (FBS, Thermo Fisher Scientific, Waltham, MA, USA). The media were free of antibiotics. Cells were cultured in a humidified incubator with 5% CO2 at 37 °C, passaged every 2–3 days, and maintained in a state of exponential growth. After centrifugation, the cells were resuspended in 0.9% saline solution for further syngeneic grafts.

### 4.3. Animal Model, Cancer Cell Inoculation and Experimental Design/Drug Treatment

Immunocompetent female BALB/c mice (VELAZ, Prague, Czech Republic) at 10 weeks of age were used in the experiments. The animals were quarantined and adapted to standard vivarium conditions with a temperature of 22 ± 2 °C, relative humidity of 45–60%, and an artificial regimen (L/D 12:12 h). During the experiment, the animals were fed with standard MP-OŠ-06 diet (Biofer, Veľký Šariš, Slovakia) and water ad libitum. Animals (*n* = 48) were randomized into five experimental groups: untreated control (C; *n* = 8); tumour control (CT26; *n* = 10); CT26 treated with IM (CT26 + IM; *n* = 10); CT26 treated with 5-FU (CT26 + 5-FU; *n* = 10); CT26 treated with IM and 5-FU (CT26 + IM + 5-FU; *n* = 10). CT26 cells (75,000 cells/mouse) were implanted subcutaneously into the mouse flanks using a 12-gauge needle after cleaning and disinfecting the skin (day 0). IM was administered intraperitoneally (i.p.) from day 5 after cell inoculation, corresponding to the onset of palpable tumours. IM was administered daily at a concentration of 0.05 IU/mouse alone or together with 5-FU, given to mice on days 5, 7, 9, 12, and 14. Then, 24 h after the final IM and/or 5-FU administration, all animals from each group were sacrificed by cervical dislocation under anesthesia.

### 4.4. Tumour Weight, Tumour Volume, and Animal Survival

During autopsy, each primary tumour was isolated, measured, and weighed on digital scales, and 5 of them were processed for histological analysis. The volume of tumours was calculated based on their diameters S1 and S2 (S1 < S2) using the formula V = π × (S1)^2^ × S2/12.

### 4.5. Blood and Serum Analysis

Blood samples were collected shortly before cervical dislocation by retro-orbital bleeding in K3EDTA tubes, 2-mL heparin-containing tubes, and 2-mL serum collection tubes (Sarstedt, Nümbrecht, Germany) and stored at 4 °C. Refrigerated samples were warmed to room temperature for 30 min before analysis. Plasma/serum tubes were placed on ice, blood for serum collection was allowed to clot for at least 30 min, and subsequently, both tubes were centrifuged at 2400× *g* at 4 °C for 15 min. Serum samples were stored at 4 °C, and biochemical analyses were performed over 2 consecutive days using an automated clinical chemistry analyzer (ELLIPSE, AMS SpA, Rome, Italy) according to the manufacturer’s instructions. Samples with whole blood in K3EDTA tubes were analyzed using an automated veterinary hematology analyzer (The Mindray BC 2800VET, Shenzhen, China).

### 4.6. Flow Cytometric Analysis of a Single-Cell Suspension from Tumour Tissue

A single cell suspension was prepared according to [68]. Briefly, mouse tumours were aseptically removed, minced into 1–2 mm^2^ pieces, and transferred into wells of a 24-well plate. Then 1 mL of collagenase solution (1 mg/mL in RPMI-1640) was added to each well and incubated for 30–60 min at 37 °C. After incubation, the contents of the wells were transferred into the cell strainer sitting in a sterile Petri dish and homogenized by using the top of a 3-mL syringe. The strainer was rinsed with 2–3 mL of medium, and obtained cells were gently passed through a 27-gauge needle (1 mL syringe) into a fresh 15-mL conical tube. After centrifugation (400 g/8 min r.t), the supernatant was discarded, and cells were resuspended in 8 mL of fresh medium. The resuspended cell suspension was filtered through another cell strainer, centrifuged (400× *g*/8 min r.t), resuspended in 5 mL of medium, and placed on ice. The viable tumour cells were counted, and 1 × 10^6^ cells of each sample were added to the bottom of a 96-well, round-bottom plate. The cells were washed by adding 200 μL of FACS buffer to each well and centrifuged at 500× *g* for 5 min at room temperature. During the centrifugations, surface staining panels were prepared in FACS buffer (Table 4). The cells were resuspended in 50 μL of the corresponding antibody mixtures and incubated for 15–20 min at 4 °C. After incubation, the cells were washed by adding 150 μL of FACS buffer to each well and centrifuged (500× *g*/5 min r.t.). Cells were then spun down, washed twice with a FACS washing solution, and resuspended in a 100 μL of fixation solution (2% paraformaldehyde in PBS and 1% BSA). Analysis was accomplished within 4 h on BD FACSAria II SORP flow cytometer (Becton Dickinson Biosciences, Piscataway, NJ, USA) using BD FACS Diva™ v 7.0 software (Becton Dickinson Biosciences, Piscataway, NJ, USA).

### 4.7. Histopathological Analysis of Tumour Sections

Tumours isolated from five animals from each experimental group were fixed in 4% paraformaldehyde in PBS (pH 7.2) for 48 h at 4 °C, washed in tap water for 5 h, and processed for preparation of paraffin sections according to the standard protocol. Tumours were embedded in low melting paraffin (Paraplast, Sigma-Aldrich), and the sections (7 µm thick) were used for a set of staining procedures. Using the standard protocol, some slides were stained with Mayer’s hematoxylin/eosin. Other slides were used to localize granulocytes after the modified Sirius Red staining protocol [69] rinsing slides in tap water. Sections were dehydrated in a set of graded alcohols and cleared in Histochoice clearing solution (Amresco, Solon, OH, USA). Finally, sections were mounted into permanent medium Histochoice mounting fluid (Amresco). Eosinophils were localized based on the presence of red-stained granules and typical nuclei, whereas neutrophils were detected based solely on the size and shape of nuclei. Enumeration of eosinophils and neutrophils in inflammatory lesions and stroma of tumours was done at 1000× magnification using Olympus Microscope BX51 and a Digital Analysis Imaging system “Analysis Docu” (Soft Imaging Systems 3.0, Prague, Czech Republic). After analysis in an average of 30 screen fields on the sections of an individual tumour, the mean number of counted cells for each section was calculated for 0.1 mm^2^ of tissue area. Finally, the mean number of the cells recorded for the sections of tumours from 5 mice was calculated, showing data for 0.1 mm^2^. The mitotic activity index was evaluated as several mitotic Figs in 10 high power fields (HPF). The area of necrosis (N/A) was usually determined based on its extent according to the size of the visual field. The smallest evaluable necrosis was its range in one HPF (0.24 mm^2^). If the necrosis was present but was less than mentioned largest magnification, it was marked as “P” (= punctiform); if necrosis was not present, the indication was “0”. The most relevant part of the CT26 tumour in paraffin block (that included the typing characteristics) with the largest representation of vital tumour epithelial component (i.e., without regressive changes such as extensive necrosis) was chosen for immunohistochemical analysis. The indirect immunohistochemical method was used for the detection of markers selected for the mechanistic study performed on whole paraffin sections with the utilization of commercially available rodent-specific antibodies (Bioss, Woburn, MA, USA; Dako, Glostrup, Denmark; Santa Cruz Biotechnology, Paso Robles, CA, USA). Immunohistochemical staining (Autostainer Link 48 /Hermes/) was performed according to the manufacturer’s recommendations. The concentration used for each primary antibody was as followed: cleaved caspase-3 1:500 (catalog no. bs-55032R); Bax 1:200 (sc-526); Bcl-2 1:200 (sc-492); Ki-67 1:50 (M7248 01). A secondary staining system (EnVision, Dual Link System-HRP, cat. No. K060911, Dako North America, Carpinteria, CA, USA) was used to visualize primary antibodies using diaminobenzidine tetrahydrochloride as a substrate. Negative controls had the primary antibody omitted. A precise morphometric method was used to evaluate the immunohistochemically detected antigen expression. Sections were screened, and Olympus BX41N was used for the microscopic analysis of digital images at magnifications of ×400. The protein expression was quantified as the average percentage of the antigen-positive area in standard fields (0.5655 mm^2^) of tumour cell hot spot areas. Three hotspots per tumour sample were analyzed using the morphometric method. QuickPHOTO MICRO software, version 3.1 (Promicra, Prague, Czech Republic), was used for the morphometric analysis of the digital images. The values were compared between treated and non-treated (control) tumour tissue specimens of mice. At least 60 tumour samples for one marker were analyzed.

### 4.8. Histopathological Analysis of Tumour Sections

All statistical analyses were performed using the Minitab version 16 software (Minitab Inc., 2013; State College, PA, USA). All data were examined for normal distribution, and appropriate tests were applied. Statistical differences between groups were analyzed using ANOVA, followed by the Tukey post hoc test or ANOVA (Kruskal-Wallis test), followed by pairwise multiple comparison procedures (Dunn’s method) or the Mann-Whitney rank-sum test. For Kaplan-Meier survival analysis, a log-rank (Mantel-Cox) test was applied. All differences were considered statistically significant when *p* < 0.05. For ethical reasons, when possible and appropriate, different tissues, organs, and whole blood samples from one animal were used for several measurements (i.e., samples to determine the different parameters in whole blood were obtained from the same animals).

## 5. Conclusions

Pre-clinical studies indicate that combinations of therapies that target distinct steps of tumour immunity may be synergistic, resulting in stronger and more sustained responses that accomplish durable tumour destruction. IM represents an attractive alternative to complement chemotherapy, which can be used to enhance the immune system after disturbances resulting from the side effects of chemotherapy. The CT26 syngeneic colorectal cancer tumour model was used to investigate the effect of IM alone or in tandem with 5-FU on colon cancer CT26 cells. The results obtained in the presented study highlight that the beneficial role of IM, claimed in previous studies, cannot be generalized to all chemotherapeutic drugs. Indeed, the 5-FU toxicity was greatly compromised when combined with IM. Moreover, the combined treatment of CT26 colorectal cancer was significantly less effective than 5-FU in slowing down tumour growth and animal survival, probably due to the increased number of TAMs and increased 5-FU cytotoxic effect related to the kidneys and liver. However, our findings have to be seen in light of some limitations. Firstly, the model we have used is based on the cancer cell line, which does not copy the evolution of the tumour as it occurs naturally. Secondly, existing pre-clinical models of human CRC that rely on syngeneic subcutaneous grafts are problematic because of increasing evidence that the immune microenvironment in subcutaneous tissue significantly differs from the gastrointestinal tract [70]. However, as shown by our study, the heterotopic syngeneic CT26 model, in addition to its use as a platform model for evaluating the efficacy of anti-cancer drug combinations, could also be used to assess phenotypic changes in the immune system [71]. Moreover, the resemblance of the CT26 models to the clinical observations of the response of CRC patients treated with immune checkpoint blockers further supports the relevance of the chosen model. Understanding the immunological events occurring in both animal models and patients undergoing chemotherapy should guide decisions about developing appropriate combinations and scheduling for the integration of chemotherapy with immunomodulators. Based on our results, IM could be further studied to find whether it synergizes with other chemotherapeutic drugs for different cancer therapies to create a more effective cancer-killing therapy.

## Figures and Tables

**Table 1:** The effect of treatments on hematological parameters in mice.

Total WBCs	C *n* = 6	CT26 *n* = 10	CT26 + IM *n* = 10	CT26 + 5-FU *n* = 10	CT26 + IM + 5-FU *n* = 10
**Total WBCs** (×10^9^/L)	11.3 ± 0.7	7.2 ± 0.6 **	7.8 ± 0.6 **	9.1 ± 0.8	8.8 ± 0.5 **
**Monocytes** (×10^9^/L)	0.30± 0.03	0.30 ± 0.04	0.40 ± 0.06	0.30 ± 0.05	0.40 ± 0.05
**Monocytes** (%)	3 ± 0	4 ± 0	5 ± 0 **	3 ± 0	4 ± 0
**Lymphocytes** (×10^9^/L)	8.8 ± 0.5	4.9 ± 0.5 ***	5.6 ± 0.5 ***	7.0 ± 0.6 ^▲▲▲^	6.3 ± 0.4 **
**Lymphocytes** (%)	78 ± 1	69 ± 2 ***	67 ± 1 ***	77 ± 2 ^▲▲▲^	74 ± 2
**Granulocytes** (×10^9^/L)	2.3 ± 0.2	2.0 ± 0.2	2.3 ± 0.2	1.8 ± 0.2	1.9 ± 0.1
**Granulocytes** (%)	19 ± 1	29 ± 2 ***	28 ± 1 ***	19 ± 1 ^▲▲▲^	22 ± 1 ^▲▲▲^
**NLR**	0.26 ± 0.03	0.44 ± 0.20 ***	0.42 ± 0.10 ***	0.25 ± 0.03 ^▲▲▲^	0.30 ± 0.06 ^▲▲▲^
**Platelets** (×10^9^/L)	729 ± 110	448.5 ± 49.5 *	279.3 ± 33.7 **^▲^	423.6 ± 67. 9 *	446.9 ± 81. 9 *
**PCT** (%)	0.5 ± 0.1	0.3 ± 0.0	0.2 ± 0.0	0.3 ± 0.1	0.3 ± 0.1
**PDW** (%)	16.0 ± 0.1	16.0 ± 0.1	16.0 ± 0.1	16.0 ± 0.1	16.0 ± 0.1
**RBC** (×10^12^/L)	13.7 ± 0.2	11.1 ± 0.2 ***	11.2 ± 0.2 ***	13.1 ± 0.5 ^▲▲^	13.0 ± 0.4 ^▲▲^
**HCT** (%)	71 ± 1	60 ± 1 ***	60 ± 0 ***	72 ± 3 ^▲▲^	73 ± 3 ^▲▲^
**HGB** (g/dL)	22.6 ± 0.4	18.2 ± 0.3 ***	18.1 ± 0.4 ***	22.1 ± 1.0 ^▲▲▲^	21.3 ± 0.7 ^▲▲▲^
**MCV** (fL)	56.7 ± 0.4	54.2 ± 0.2 **	53.8 ± 0.4 **	55.2 ± 0.4 **	55.9 ± 0.6 ^▲▲^
**RDW** (%)	15.7 ± 0.2	15.6 ± 0.1	15.3 ± 0.2	15.8 ± 0.1	15.5 ± 0.2
**MCHC** (g/L)	289.6 ± 2.1	303.1 ± 1.1 ***	299.2 ± 1. 9 ***	305.7 ± 2.0 ***	293.3 ± 3.6 ^▲▲▲^
**MPV** (fL)	7.1 ± 0.2	7.4 ± 0.1	7.3 ± 0.2	7.7 ± 0.2	7.3 ± 0.2

Data are expressed as mean ± SD. * *p* < 0.05, ** *p* < 0.01, *** *p* < 0.001 as compared to INT; ▲ *p* < 0.05, ▲▲ *p* < 0.01, ▲▲▲ *p* < 0.001 as compared to CT26; C- healthy control; CT26-tumour control; CT26 + IM-CT26 treated with IM; CT26 + 5-FU-CT26 treated with 5-FU; CT26 + IM + 5-FU-CT26 treated with IM and 5-FU. WBC (White Blood Cells); RBC (Red Blood Cells); NLR (Neutrophil-Lymphocyte Ratio); HGB (Haemoglobin); HCT (Haematocrit); PCT (Plateletcrit); MCV (Mean Corpuscular Volume); RDW (Red Cell Distribution Width); MCHC (Mean Corpuscular Haemoglobin Concentration); PDW (Platelet Distribution Width); MPV (Mean Platelet Volume).

**Table 2:** The effect of treatments on biochemical parameters in mouse serum samples.

	C A	CT26 B	CT26 + IM C	CT26 + 5-FU D	CT26 + IM + 5-FU E	Statistical Significance
**Total CH** (mmol/l)	4.24 ± 0.05	4.04 ± 0.10	4.42 ± 0.08	4.33 ± 0.09	4.65 ± 0.03	B vs. E *** A vs. E *
**LDL-CH** (mmol/L)	0.39 ± 0.01	1.40 ± 0.06	0.52 ± 0.04	0.48 ± 0.02	0.51 ± 0.02	B vs. A, C, D, E *** A vs. C, E *
**HDL-CH** (mmol/L)	0.99± 0.03	1.84 ± 0.02	1.90 ± 0.07	2.15 ± 0.07	2.11 ± 0.02	A vs. B, C, D, E ***
***TGC*** (mmol/L)	2.24 ± 0.02	2.95 ± 0.19	3.35 ± 0.34	2.29 ± 0.04	2.35 ± 0.09	B vs. A, D * Cvs. A, D, E ***
***AST*** (μkat/L)	2.15 ± 0.34	3.98 ± 0.07	3.82 ± 0.09	3.06 ± 0.19	3.78 ± 0.02	B vs. D * A vs. B, C, E *** D vs. A *
***ALT*** (μkat/L)	1.66 ± 0.01	3.24 ± 0.99	2.23± 0.34	1.92 ± 0.49	2.27 ± 0.09	A vs. E ** D vs. E *
***ALP*** (μkat/L)	3.05 ± 0.03	2.35 ± 0.31	2.44 ± 0.06	2.51 ± 0.13	2.41 ± 0.15	A vs. C ** A vs. B, D, E *
**Total-P** (g/L)	62.60 ± 0.32	60.27 ± 4.81	66.80 ± 2.96	56.20 ± 4.43	63.53 ± 3.67	NS
**Urea** (mmol/L)	5.74 ± 0.09	6.46 ± 0.01	7.46 ± 0.41	6.66 ± 0.21	8.21 ± 0.32	B vs. E ** A vs. C ** D vs. E ** A vs. E ***
**Albumin** (g/L)	32.90 ± 0.35	31.13 ± 1.18	32.87 ± 0.29	32.60 ± 0.10	32.40 ± 0.91	NS
***Creatinine*** (µmol/L)	27.00 ± 1.15	43.67 ± 1.76	34.00 ± 0.57	21.00 ± 1.08	33.75 ± 1.03	B vs. A, D *** C vs. D ** D vs. E **

Data are expressed as mean ± SD; * *p* < 0.05, ** *p* < 0.01, *** *p* < 0.001; C-healthy control; CT26-tumour control (*n* = 10); CT26 + IM-CT26 treated with IM (*n* = 10); CT26 + FU-CT26 treated with 5-FU (*n* = 10); CT26 + IM + FU-CT26 treated with IM and 5-FU (*n* = 10). *** *p* < 0.001 vs. CT26 group. AST- aspartate aminotransferase; ALT-alanine aminotransferase; ALP-alkaline phosphatase; TGC-triglycerides. CH-cholesterol; *p*-Proteins.

**Table 3:** The immunohistochemical (IHC) evaluation of caspase-3 (cytoplasmic), Bax, Bcl-2, and Ki67 expressions and histopathological evaluation of mitotic activity index (MAI) in mouse CT26 colon carcinoma cells after the administration of IM, 5-FU, and combination of IM + 5-FU.

	CT26	CT26 + IM	CT26 + 5-FU	CT26 + IM + 5-FU
Caspase-3	26.33 ± 0.90	28.22 ± 1.13	29.36 ± 1.07 ^a^	28.99 ± 0.89 ^a^
Bax	14.37 ± 0.80	16.60 ± 0.84	19.57 ± 0.96 ^aaa,b^	19.78 ± 1.06 ^aaa,b^
Bcl-2	33.24 ± 1.34	29.36 ± 1.22 ^a^	27.21 ± 1.27 ^aa^	23.68 ± 1.59 ^aaa,bb^
Bax/Bcl-2	0.45 ± 0.04	0.58 ± 0.04 ^a^	0.73 ± 0.04 ^aaa,b^	0.92 ± 0.10 ^aaa,bb^
Ki-67	15.77 ± 0.73	15.02 ± 0.68	15.18 ± 1.13	15.17 ± 0.83
MAI	4.03 ± 0.24	4.13 ± 0.25	3.80 ± 0.53	3.48 ± 0.26

Data are expressed as means ± S.E.M. Significantly different, ^a^ *p* < 0.05, ^aa^ *p* < 0.01, ^aaa^ *p* < 0.001 vs. CT26, ^b^ *p* < 0.05, ^bb^ *p* < 0.01 vs. CT26 + IM. Data from IHC analysis represent the expression of proteins quantified as the average percentage of antigen-positive area in standard fields (0.5655 mm^2^) of tumour hot spot areas. At least 60 images for one marker were analyzed. MAI represents the number of mitoses per ten high power fields (×400) in each evaluated tumour.

**Table 4:** Immune cell populations based on the expression of cell surface markers [12] and specification of used anti-mouse monoclonal antibodies (eBioscience).

Immune Cells	Cell Markers	Fluorochrome	Panel
Leukocytes	CD45+	FITC	F4/80 (1:100)
T-Lymphocytes	CD45+ CD3+	PE	CD49b (1:100)
B- Lymphocytes	CD45+ CD19+	PerCP-Cy5.5	CD3 (1:100)
Macrophages	CD45+ F4/80+	APC	CD19 (1:200)
